# Determining factors in the retention of physicians in rural and underdeveloped areas: a systematic review

**DOI:** 10.1186/s12875-020-01279-7

**Published:** 2020-10-23

**Authors:** Nasrin Mohammadiaghdam, Leila Doshmangir, Javad Babaie, Roghayeh Khabiri, Koen Ponnet

**Affiliations:** 1 Department of Health Policy& Management, Tabriz Health Services Management Research Center, Iranian Center of Excellence in Health Management, Tabriz University of Medical Sciences, Tabriz, Iran; 2 Social Determinants of Health Services Research, Health Management and safety Promotion Research Institute, Tabriz University of Medical Sciences, Tabriz, Iran; 3 Faculty of Social Sciences, imec-mict-Ghent University, Ghent, Belgium

**Keywords:** Health care workers, Health system, Physicians, Retention, Rural areas, Under developed areas

## Abstract

**Background:**

Imbalance in distribution of Health Care Workers (HCWs) in a country is a global challenge. Almost all of the rural and underdeveloped areas are struggling with the shortage of HCWs, especially physicians. Therefore, this study aimed to identify factors governing the retention of physicians in rural and underdeveloped areas.

**Methods:**

International databases including Scopus, PubMed, Web of Science, Proquest, and Embase were searched using Mesh terms in order to find peer-reviewed journal articles addressing physicians’ retention factors in rural and underdeveloped areas. The records were screened, and any duplicate results were removed. The quality of the studies was assessed according to the Critical Appraisal Skills Program developed for different types of studies. Then, through content analysis, the related factors were identified from finally selected papers, coded, and categorized.

**Results:**

The initial search resulted in 2312 relevant articles. On the basis of specific selection criteria, 35 full-text articles were finally reviewed.. The major affecting factors in physicians’ retention in rural and underdeveloped regions were classified into the following six categories: 1) financial; 2) career and professional; 3) working conditions; 4) personal; 5) cultural; and 6) living conditions factors.

**Conclusion:**

There is a complex interplay of factors governing physicians’ retention in rural and underdeveloped areas. If health organizations are concerned with physicians’ retention in deprived areas, they should take into account these main factors. Moreover, they should develop policies and strategies to attract and retain physicians in rural and underdeveloped areas.

## Background

Health Care Workers (HCWs) are the backbone of health organizations [1] and are considered as the main factor in public access to health services [2, 3]. In most countries, 10% of the total government employment is allocated to the health sector [4]. The World Health Organization (WHO) defines Health Workers (HWs) as all the people who are engaged in actions primarily intended to improve health [5]. The imbalance in the distribution of HCWs within countries is a common serious problem and can lead to challenges such as shortage of physicians in underdeveloped areas which in turn could pose challenges for the public access to health care services [6, 7].

Physicians play a vital role among the HCWs [4]. Although they form a major group of HCWs, they have been neglected in the discussions about the health system functions in many developing as well as developed countries [8]. Migration of physicians from rural and underdeveloped areas to affluent ones has caused major concerns for health policymakers. A disproportionate distribution of physicians can cause a disturbance to the performance and stability of health systems [9]. In most of the rural and underdeveloped areas, lack of physicians is a long-standing challenge with serious consequences for the quality and quantity of provided services and equity of access [10]. Moreover, it restricts access to health services for people in these areas.

Previous experiences suggest that, in general, increasing the number of physicians in a country is not a determining factor in the increase in their retention rate in rural and underdeveloped areas [11, 12].

Conducted in 1998 in the United States, one study revealed that there was a 75% shortage of physicians in rural areas [13]. The results of a similar study in Argentina in 2014 showed that 21% of the physicians had a strong desire to leave the deprived areas, 57.3% had a moderate desire to desert these areas, and 21.5% were reluctant to leave the aforementioned areas and were willing to be active in their relevant areas [12]. In Iran, the distribution of HCWs is not equitable between and within provinces, especially in deprived and underdeveloped areas [14, 15]. Based on a study conducted in Kerman in 2014, the proportion of doctors who had quit working in deprived and rural areas was 26, and 77.3% of them intended to leave health services in the near future [16].

Many countries suffer from critical shortage of physicians in marginal and underdeveloped rural areas. Moreover, little is known on determining factors in the retention of physicians in these areas. Therefore, this study aims to identify pivotal factors governing physicians’ retentions in rural and underdeveloped areas in order to develop evidence-informed policy interventions to deal with the issue of physicians’ inaccessibility.

## Methods

### Search strategy

A systematic review of the literature was conducted in order to identify the factors that influence physicians’ retention in rural and underdeveloped areas. We searched five distinct databases including Scopus, Web of Science, PubMed, Embase, and ProQuest. The search focused on the articles which had been published in the relevant databases from their inception until December, 2019. The reference lists of the articles were also checked to find additional studies to complete the search process and to find all of the relevant studies. The detailed search terms were developed in consultation with a librarian. The search terms and the sample of search strategy, taken from the PubMed database, are provided in Table 1 in the Additional file 1.

### Study selection process

All of the retrieved citations were imported to EndNote (V. X8; Clarivate Analytics, Philadelphia, PA) after searching in the relevant databases. The title and abstract (if available) of each unique citation were screened by one reviewer (NM) according to the prespecified inclusion and exclusion criteria. The final stage of the screening of the articles (full-text review) was carried out by the two researchers (NM and LD). The researchers (NM and LD) independently extracted relevant data from the articles. In cases of disagreements, the researchers’ views on the elimination of the studies were compared in order to reach a consensus. There were few discrepancies in the data extractors’ views.

### Inclusion and exclusion criteria

On the basis of the inclusion criteria, the selected studies: 1) were published in English language; 2) focused on physicians, including general practitioners, general physicians, family physicians and specialists as the target population; 3) were related to deprived, remote, rural and underdeveloped areas; 4) involved any type of article including letters to the editor, any type of review, original articles, debates, and perspectives; and 5) were full-text articles.

Moreover, based on the exclusion criteria, the excluded articles were: 1) the ones whose target populations were not physicians for example they were administrative staff, managers, or nurses in the health sector; 2) full-text articles were not available.; and 3) were low quality based on the quality appraisal analyses.

### Data extraction

In order to extract the relevant data, a data extraction checklist was used. Title, author(s), referencing style, country, year of publication, study type, study population, data collection method, extracted factors, quality appraisal, effect of the context on how the factors influenced the retention of physicians in rural and underdeveloped areas, and the positive or negative effects of the extracted factors on the retention of physicians were noted. In addition, the checklist contained a column for presenting research findings which were related to the factors governing the retention of physicians in the underdeveloped areas and their effects.

### Quality appraisal

The Critical Appraisal Skills Program (CASP) checklists were used to assess the quality of the articles. CASP checklists are quick, simple, and straightforward tools for answering common questions on a scientific study [17]. We downloaded various relevant standard checklists from the website of the program (www.casp-uk.net) for the various types of studies (i.e., cross-sectional, review, qualitative, and analytical) which were included in our study.

The used checklists included 10 questions on the aims of the research, appropriateness of the methodology, research design, data collection, and data analysis. In order to assess the quality of studies, each question was scored 1 point for *Yes* or 0 for *No*. Then, the studies were categorized as *low quality* (i.e., studies with a score range of 0 to 3), *medium quality* (i.e., studies with a score range of 4 to 6), and *high quality* (i.e., studies with a score range of 7 to 10). Two of the researchers conducted quality appraisal independently. Finally, the differences in the researchers’ appraisals were resolved by discussion.

### Data analysis

Inductive content analysis was performed in order to understand, extract, and categorize factors governing the retention of physicians in rural and underdeveloped regions. Two members of the study team coded and categorized the data. To this end, they: 1) examined the selected full-text studies carefully and extracted the determining factors, 2) developed potential themes which involved the extracted factors, 3) generated a thematic map, 4) gave a name to the themes and defined and generated examples for each of them, and 5) assessed the reliability of the analyses through discussions regarding the contradictions to achieve full agreement.

## Results

**Fig. 1:**
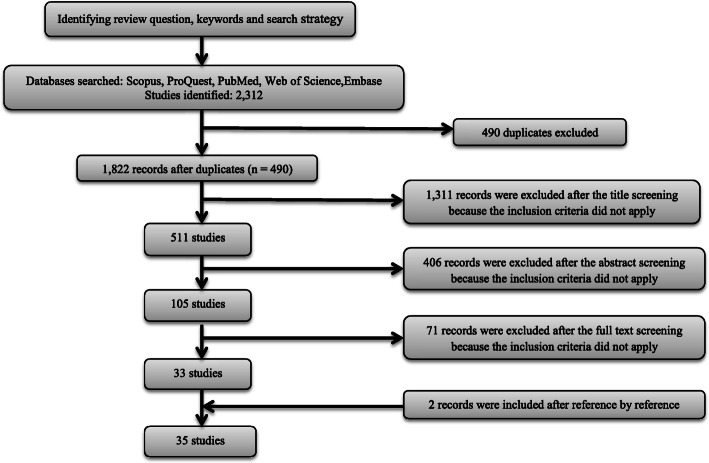
Flow chart of study selection process

The searching process from the five databases provided the researchers with a total of 2312 articles: (1737 in Scopus, 289 in Embase, 215 in PubMed, 62 in Proquest, and 9 in ISI Web of Science,). A total of 490 duplicate articles were identified, leaving us a total of 1822 unique citations on the topic. The screening process of the extracted studies is shown in Fig. 1.

Moreover, 1311 titles were excluded due to the fact that they were not relevant to the purpose of study. The abstracts of 511 remaining studies were reviewed. Furthermore, 406 studies, whose aims were different from our purpose, were excluded. The remaining 105 full-text studies were carefully examined. On the basis of this examination, 71 studies were excluded because they only described the distribution of physicians and their desertion rates. In addition, these studies did not identify factors associated with retention or desertion. However, two related studies were identified using a manual search (reference by reference) and were added to the study. At the end of this phase, 36 studies were included in the present study. One article did not get an acceptable quality score and was excluded from the corpus. A careful examination of the selected articles showed that 45 and 55% of the studies were concerned with the exploration of the factors governing the retention of physicians in underserved settings of the developed countries and the developing or underdeveloped countries, respectively. The characteristics and findings of all included studies are presented in Table 1.

**Table 1:** The characteristics and findings of included studies in the review

No	Title and Authors (Reference No)	Country and Date	Type of study	Study population	Data collection method	Quality appraisal	Effect of the context on how the factors influence the retention of physicians in rural and underdeveloped areas
1	Recruitment and Retention of General Practitioners in Rural Canada and Australia (Marco Viscomi, Hon BSc) [18]	2013 Canada and Australia	review	*n* = 86 sources for review/ Canada Astralia	MEDLINE/Ovid	High	-Further follow-up of the highlighted educational programs is warranted to ensure continued refinement of socially accountable policies and initiatives.
2	Sustaining Family Physicians in Urban Underserved Settings (Anne Getzin MD, BennIe L) [19]	2016 United States	qualitative study	*N* = 16family physicians	Semi-Structured Interviews/multi-step process	High	-Training in the personal and professional skill sets identified may improve physician retention in urban underserved settings.
3	Retention of General Practitioners in Rural Nepal: A qualitative study (Katrina Butterworth, Bruce Hayes) [20]	2008 Nepal	qualitative study	n = 86 General practitioner Questionnaire *n* = 11 Semi structured interviews	Questionnaire/ semi structured interview and focus group discussions	High	-Career development is considered as a key issue that must be addressed by the government of Nepal. GPs need to have a clear career ladder, with recognition of the value of service in rural areas. -A multifaceted, holistic response is necessary. From the level of community awareness, a career structure and financial remuneration to adequately set up hospitals, functional teams, family support, continuing professional development and a secure working environment – each area must be addressed for the whole to function.
4	Factors Affecting Leave out of General Practitioners from Rural Family Physician Program( Amiresmaili M, Khosravi S, YazdiFeyzabadi V) [16]	2014 Kerman, Iran	Survey cross-sectional	*n* = 271 Family Physicians	Questionnaire	High	-Educating native manpower has been one of the main strategies of medical education system in Iran in order to increase physicians’ retention in deprived areas. -General practitioners cooperate with the educational programs as part time physicians and seek further education to gain a competitive advantage and better social position and income.
5	Analysis of a survey on young doctors’ willingness to work in rural Hungry (Edmond Girasek) [9]	2010 Hungary	Survey	*n* = 785 four Hungarian medical universities	Questionnaires and focus group interviews		-The current system of medical training in Hungary tends to produce doctors who want to live in big cities and work in central hospitals. Rural regions and non-in-patient service alternatives seem either not to be targeted or seen as unattractive work places. More doctors would be willing to work in smaller towns and villages if in-hospital training was altered and if doctors were offered adequate incentives as part of a comprehensive human resource strategy (high salaries, high professional standards, good working environment, reasonable workload). If these changes do not occur, the existing geographical and structural imbalances will not be improved.
6	Rural Physician Satisfaction: Its Sources and Relationship to Retention (Donald E. Pathman, Eric S. Williams, Thomas R. Konrad) [14]	1996 United States	Survey/ Cross-sectional	*n* = 620 prima y care physicians	Mail questionnaires	High	-Physicians’ satisfaction with their communities and their opportunities to achieve professional goals predicted longer retention. Satisfaction with income also tended to predict longer retention.
7	Retention of Primary Care Physicians in Rural Health Professional Shortage Areas (Donald E. Pathman, MD, MPH, Thomas R. Konrad, PhD, Rebekkah Dann, MS, and Gary Koch, PhD) [21]	1991 United States	Survey/ Cross-sectional	*n* = 505 physicians/ randomly selected	Questionnaire	High	-Retention is related to modifiable characteristics of work, whereas recruitment is related to the relatively immutable characteristics of physicians’ backgrounds and professional and lifestyle preferences, as well as the socioeconomic features of communities. -To promote retention, local, state, and federal programs can promote practice ownership through low-interest loans and start-up income guarantees. Practice administrators can build a sense of personal investment and control among employed physicians by offering leadership opportunities and providing a greater voice in clinic policies and work schedules. -Local hospitals and practice networks can reduce on-call frequency by coordinating cross-coverage arrangements. Work demands while on-call can be lessened by providing telephone call triage systems and full-time physician staffing in local emergency rooms.
8	Why do Junior doctors not want to work in a Rural location and What would induce them to do so? (Mary E. Rogers, Judy Searle and Peter A. Creed) [22]	2009 Australia	Survey/ Cross-sectional	*n* = 190 Junior doctors	Questionnaire	High	-Infrastructure and professional development opportunities are the influential factors. -Women gave more importance to partner and family factors than men.
9	What Factors Contribute most to The Retention of General Practitioners in Rural and Remote areas? (Deborah J. Russell, Matthew R. McGrailB,, John S. Humphreys, and John WakermanB,) [23]	2008 Australia	Survey/ Cross-sectional	*n* = 1189 general Practice	Questionnaire	High	-Ideally such factors would be supported by an existing evidence base, be amenable to measurement and able to be influenced by management policy (such as remuneration, availability of suitable housing, paid locum relief, work culture and perceptions of work life balance). Such information may facilitate the further ‘unpacking’ of factors that have been demonstrated to be important for Australian rural and remote GP retention.
10	Physician Shortages in Rural Vietnam: Using a labor market approach to inform policy. (Marko Vujicic, Bakhuti Shengelia,, Marco Alfano, Ha Bui Thu) [7]	2009–2010 Vietnam	Survey	*n* = 292 Physicians (rural *n* = 57 and urban *n* = 235)	Questionnaire/ multistage sampling	High	-Providing preferential access to short term training and paying financial bonuses in rural areas are policies that the government of Vietnam should seriously consider. For short-term training, the government might consider a program where physicians in rural areas are guaranteed access to short-term training courses that are most in demand. In terms of bonuses, these can be implemented either as separate allowances or by revising the current salary points system to reward locating in a rural area much more.
11	Factors and Outcomes in Primary Care Physician Retention in Rural areas (Glasser M, PhD MacDowell M, DrPH, MBA Hunsaker M, MD Salafsky B, PhD Nielsen K, MPH Peters K, DrPH Meurer M, MS) [13]	1997 United States	Survey	*n* = 107 Primary care and Specialty care	Non structured interview	*Medium*	-Keys to success in rural physician retention seem to include identifying and recruiting medical students of rural origin and focusing on a healthy practice environment. Policy makers need to work with local government; schools and employers to offer programmers that provide information on health careers in rural areas and begin to identify local youth for induction in rural health care.
12	Relationship Between Personal Characteristics of Specialist Physicians and Choice of Practice Location. (Taati Keley E, Ravaghi H, Salehi M, Nasiripour A, Abdi Z, Meshkini A) [24]	2013 Iran	Survey/cross-sectional	*n* = 3825 physicians who graduated from all public medical schools across the country between 2009 and 2012		High	-Increasing the enrollment of physicians with a rural background in residency programs may solve the problem of uneven distribution of specialist physicians in Iran. Because female physicians are less willing to work in the underdeveloped areas than male physicians, increasing the number of male student admissions to residency programs, particularly in certain specialties that are more in demand in the underdeveloped areas, could alleviate the problem of uneven distribution of physicians in the short run. Further, programs that support raising the admissions of female students with a rural background into local medical universities along with providing incentives to encourage them to live and work in rural areas should be put on the policy agenda.
13	Attracting and Retaining Doctors in Rural Nepal (Shankar PR) [8]	2010 Nepal	Review	Original research articles, reviews, magazine articles and project reports dealing with Nepal and other developing countries during the period 1995 to 2010	PubMed/Google scholar/ WHO’s HINARI database	*Medium*	-A range of strategies developed elsewhere could be used in Nepal, especially community-oriented medical education that involves rural doctors in training medical students. The reimbursement of tuition fees, assistance with relocation, and provision of opportunities for academic and professional advancement for rural doctors should also be considered. Government investment in improving working conditions in rural Nepal would assist rural communities to attract and retain doctors.
14	Factors affecting the work of physicians in rural areas of Turkey (Mollahaliloglu S, Ugurluoglu Ö) [25]	2009 Turkey	Survey	*n* = 1340 physicians working in urban areas	Questionnaire	High	-Medical residents working in the university hospitals, their willingness to work in rural areas can be related to their continuing education with temporary status and their obligation to go to another healthcare institution for a long-term employment option. Physicians who are in the early stages of their careers can be thought to be more open to changing location compared with more experienced ones who have an established career path. -Financial incentives, non-monetary incentive policies that consider the physicians’ views should be developed to achieve permanent success. -Regulations that make the physician’s life easier, such as housing provision, better service infrastructure and flexible working regimes, can motivate young and single physicians to work in rural areas.
15	Factors Affecting Willingness to Practice Medicine in Underserved Areas (Borracci RA, Arribalzaga EB) [12]	2013–2014 Argentina	Survey/ cross-sectional descriptive	*n* = 400 Argentine Medical students	Questionnaire	High	-Government policy-makers must recommend changes in resource allocation to better promote official proposals and opportunities to work.
16	Factors Influencing the Geographic Distribution of Physicians (Ravaghi H, Taati E, Abdi Z) [26]	2012 Iran	qualitative study	*n* = 82 key officials from medical universities	Open-ended Questionnaire	High	-Policies such as providing more financial and non-financial incentives, reducing disparities between physicians’ income in rural and metropolitan areas, selection of students with rural background, and supportive measures for physicians working in underserved areas were recommended.
17	Key factors leading to reduced Recruitment and Retention of Health Professionals in Remote areas of Ghana (Rachel Cnow, KwesiAsabir) [27]	2010 Ghana	qualitative study	*n* = 84 doctors and medical leaders	In-depth interviews	High	-Expanding opportunities for post-graduate specialization may offer a significant return on investment; establishment of postgraduate training in Obstetrics and Gynecology in Ghana in 1989 led to high retention rates among graduates of the program. Graduates cited the appeal of adding a chance for specialization in their own country to their continued service in Ghana. -Career advancement incentives will be critical to any successful incentive package. Proposed incentives include guaranteed promotion or study opportunity after service in hardship areas contact with mentors through rural rotation of specialists or remote learning centers, and reliable terms of appointment with fixed end-points.
18	What Factors Influence the Choice of Urban or Rural Location for Future Practice of Nepalese Medical Students? (Bhim Prasad Sapkota, Archana Amatya) [28]	2015 Nepal	Cross-sectional descriptive	*n* = 393 medical students from four medical colleges	Questionnaire/ In-depth structured interviews	High	-The government should attract the students from a rural place of rearing and rural secondary education for medical education. -Newly established medical college by the Nepal government (Patan Academy of Health Sciences) has started to enroll students having a rural rearing and rural schooling in MBBS.
19	A systematic review of strategies to recruit and retain primary care doctors (Puja Verma, Arabella Stuart) [29]	January 2015 England	review	*n* = 42 studies	MEDLINE, EMBASE, CENTRAL	High	
20	Factors That Influence the Turnover Intention of Chinese village Doctors based on the Investigation Results of Xiangyang City in Hubei Province (Pengqian Fang, Xiangli Liu) [30]	July and August in 2012 China	Survey	*n* = 1889 Doctors	Questionnaire	High	-The government should raise the income of village doctors and provide them with suitable promotion opportunities and security insurance, such as health insurance and pension insurance. In addition, low education levels of village doctors, as a barrier to achieve the educational goals for the village doctors set by the government, should also be tackled through further on the-job training or by recruiting more college medical graduates into the village clinics.
21	Physicians’ retention rate and its effective factors in the Islamic Republic of Iran (Ehsani-Chimeh E, Majdzadeh R, Delavari S, et al) [31]	2000–2001 Iran	Survey /cross-sectional	*n* = 5482 physicians	Questionnaire	High	-About three-fourths of Iranian physicians would work in underserved areas if there were some special privileges for them, mainly income and employment relationship. Younger males and those who belonged to the Medical Student Boom Generations had more inclination.
22	Assessment on causes of physicians’ abdication from Rural Family Physician Plan in 2012 (Sadighi S, Amini M, Pourreza A) [32]	2012 Iran	Analytical descriptive study	*n* = 26 Family physicians	Questionnaires	High	-With regard to the effective components in social and cultural resignation and leave family physicians in the main step of implementing this national plan, optimally between the two sides in a suitable provider of service and recipient country according to current laws will be.
23	Factors influencing desertion of family physicians working in rural areas with deprivation index less than 1.4 (prosperous) in 2010 (Atefi A, Aghamohammadi S) [33]	2010 Iran	cross-sectional descriptive	*n* = 6618 Family physicians	Questionnaire/ interviews	High	-There was an increasing trend on dropping out of family physician program. To seize this trend, some actions could be done, including: to promote authorization for hiring family physicians, to reform the amount of payments, to ensure timely payments, to develop residency program on family physicians and dedicate an admission priority to active family physicians to enroll in the program, and finally to set a higher salary for family physicians practicing in rural areas.
24	Factors Influencing Retention of Rural Pennsylvania Family Physicians. Esther M, Forti, Kenneth E. Martin, Robert L. Jones, and Herman, J [34]	1993 United State	Cross-sectional descriptive	*n* = 398 Family Physicians	Questionnaire/ Mail Survey	High	-Health system should use the strategies that minimize perceptions of professional isolation and policy efforts that address reimbursement differentials and compliance issues in order to minimize many complaints of rural family physicians.
25	Determining the Causes of Discontinuation of Family Physicians Working in Mashhad University of Medical Sciences (Ehsan Mosa Farkhany, Hosein Khooban, Behruz Dahrazama, Vahid Reza Arefi,Fariba Saadati) [35]	2012 Iran	Analytical study	*n* = 156 Family physicians	Questionnaire	High	-The Ministry of Health and Medical Education should focus on increasing annual per capita and credit The Rural Family Physician Program has taken serious action and also revised the plan to reduce the responsibilities of members of the health team.
26	Physician preferences for working in deprived areas: a systematic review of discrete choice experiment(Hamouzadeh P, Akbarisari A, Olyaeemanesh A, Yekaninejad MS) [36])	2017 Iran	Systematic review	*n* = 14 studies	PubMed, Embase, Web of Science Core Collection	High	-Financial attributes are not the only significant attributes considered by the physicians for deciding where to practice, but also the other non-financial attributes are important. It is suggested that based on the economic, social and cultural conditions of each country, a specific incentive package, including a set of financial and non-financial incentives, is developed to attract physicians to the deprived areas.
27	Factors influencing turnover intention among primary care doctors: a cross-sectional study in Chongqing, China (Tong Wen, Yan Zhang) [37]	2013 China	a cross-sectional study	*n* = 440 doctors	Interviewed	High	-Improving job satisfaction, in terms of salary, promotion and job safety, is crucial for reducing turnover intention among primary care doctors. Therefore, we suggest that the government increase its financial investment in primary care facilities, especially in less-developed areas, and reform incentive mechanisms to improve the job satisfaction of primary care doctors. The government should consider policies such as establishing a social pension programme for village-level doctors and providing more opportunities for job promotion among primary care doctors, especially township-level doctors.
28	The role of rural communities in the recruitment and retention of women physicians (Paladine HL, et al) [38]	2019 United States	Qualitative study	*n* = 25 women family physicians	Interview	High	-Resident selection based on a predisposition to work in underserviced areas, related to either interest or family ties, and immersing them in family practice in these areas, can enhance recruitment and retention in underserviced areas. -Positive residency experiences, established relationships with local specialists, health professionals and community services, and opportunities to practice as they had trained and how they preferred to practice, contributed to physicians’ decision to practice in the same geographical area in which they train.
29	Keeping family physicians in rural practice (JTB Rourke) [39]	2003 Canada	Cross-sectional mailed survey	*n* = 276 physicians and 210 residents	Survey questionnaire	High	-It is important to facilitate referrals and provide specialist support -Availability of locums can make a big difference in continuing patient care and allowing time off for CME, family holiday time, maternity leave, and so on. -A comprehensive package based on highly rated solu- tions is more likely to be successful than politically expedient measures.
30	Retaining Doctors in Rural Bangladesh: A Policy Analysis (Taufique Jourder et al) [40]	2018 Bangladesh	Qualitative study	n = 11 relevant policy elites	Group discussions	High	-Applicants with relevant expertise to be recruited; recruitment should be quick, customized, and transparent; career tracks (General Health Service, Medical Teaching, Health Administration) must be clearly defined, distinct, and respected. -Facilities must be ensured prior to postings, female doctors should be prioritized to stay with the spouse, field bureaucrats should receive non-practicing allowance in exchange of strict monitoring, and no political interference in compulsory service is assured. - Specific policy guidelines should be developed to establish rural medical colleges. -Commitment from the highest level of political hierarchy is the key to the successful implementation of the rural retention policies of the government.
31	Physician recruitment and retention in Manitoba: results from a survey of physicians’ preferences for rural job (Witt, Julia) [41]	2017 Canada	survey	*N* = 561 physicians	Questionnaires	High	-Several of the attributes that reflected the need for professional and social inclusion were found to be important: group practice, community incentives (during the first year) and access to clinic technology, particularly telehealth.
32	Recruitment and retention of physicians in rural Alberta: The spousal perspective (Myroniuk, L., Adamiak, P., Bajaj, S., Myhre, D.L.) [42]	2016 Canada	Qualitative study	N = 84 physicians	Semi-structured interviews	High	-Considerations to accommodate the educational, professional and cultural needs of the physician spouse must be highlighted in policy if large areas of underserved rural communities continue to rely on international recruitment. -Leveraging new technologies to provide online access to education, jobs and connection to family and friends may offer a solution to some of the challenges faced by spouses of physicians that practice medicine rurally.
33	Physician recruitment and retention in rural and underserved areas (Lee DM, Nochols T) [43]	2014 Canada and USA	review	n = 86 sources for review/ Canada USA	Academic Search Complete, PubMed and The Cochrane Collaboration	High	-The first strategy is to get the chief executive officer (CEO) involved in physician recruitment, who must: first, develop a recruiting team and meet the team at least quarterly to monitor progress and offer guidance; second, participate in developing marketing and recruiting strategies; third, interview all promising candidates; fourth, financially support the recruiting team’s recommended financial incentives for physicians; fifth, hold the recruiting team members accountable by tying their compensation and advancement in the organization to their recruiting performance; and sixth, request the recruiting team to brief candidates regarding abrasive personalities on the existing medical staff.
34	The role of distributed education in recruitment and retention of family physicians (Lee, J; Walus, A; Billing, R; Hillier, LM) [44]	2016 Canada	Qualitative Study	*N* = 32 family physicians who graduated from a DME residency training programme.	Semi structured in person interviews	High	-Resident selection based on a predisposition to work in underserviced areas, related to either interest or family ties, and immersing them in family practice in these areas, can enhance recruitment and retention in underserviced areas. -Positive residency experiences, established relationships with local specialists, health professionals and community services, and opportunities to practice as they had trained and how they preferred to practice, contributed to physicians’ decision to practice in the same geographical area in which they train.
35	Rural physician supply and retention: factors in the Canadian context (Fleming, Patrick; Sinnot, Mari-Lynne) [45]	2018 Canada	Review	n = 42 studies	PubMed, Embase, CINAHL and ERIC	High	-Overall strategies to improve retention will ensure a stable physician supply and, therefore, will have benefits for population health over the long term. Locally trained physicians practice longer in their home province than out-of-province graduates and international medical graduates. It is important to ensure that there are rural educational opportunities for learners in undergraduate and postgraduate medical training.31 ultimately, enhanced, forward-thinking retention strategies will improve community health and help correct rural disparities for Canadians.

The identified themes were classified into six main categories of factors: financial, career and professional, working conditions, personal, living conditions, and cultural.

Financial factors focused on the physicians’ income level, salary, payment interval, reimbursement of tuition fees, various allowances and loan repayment. Personal factors were related to the physicians’ demographic factors such as age, sex, education level, marital status, number of children, rural background, previous exposure or service in a deprived area and birthplace. Career and professional factors included factors such as job performance, evaluation of superiors, job improvement and educational opportunities, job position, willingness to change the performed job or the employer or support for professional development or research marketing and opportunities to achieve professional goals.

Working conditions or environment referred to the employment relationships and comprised all of the existing circumstances which affected physicians’ work in the workplace, including work schedules, breaks, work hours, legal rights, and responsibilities, possibility of relocation and job security and job flexibility. Living conditions or recruitment situation encompassed the conditions in which physicians lived such as suitable housing and accommodation, educational opportunities for the children, access to refreshment facilities as well as recreational activities. Finally, cultural factors involved the customs, traditions, beliefs, moral values, code of communication and used language(s) of the rural community.

**Fig. 2:**
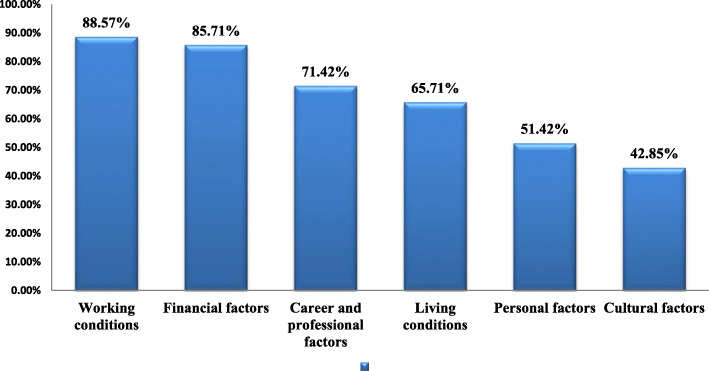
Percentage of the influential factors in the selected studies

Table 2 provides the influential factors in the retention of physicians in rural and underdeveloped areas based on the identified category of factors. Moreover, Fig. 2 provides information on the percentage of the influential factors in the selected studies.

**Table 2:** Influential factors on retention of physicians in rural and underdeveloped areas

Category	Positive factors	Negative factors	Not clear/Not reported
**Financial factors**	➢ Improved earning potential (18) ➢ Individual incentive programs (18) ➢ Financial incentives (20, 23, 18, 25, 27, 29, 41, 43, 45) ➢ Adequate and fair pay scale (20) ➢ Transparency of the payment system (16) ➢ High salaries (9, 24) ➢ High Earnings from the practice (9) ➢ Bolstering incomes for employed physicians (14) ➢ Favorable salary levels (14) ➢ Increasing reimbursement levels (14) Remuneration (12,18, 23, 27) ➢ Desirable average monthly payments (7) ➢ Various allowances (7) ➢ Financial bonus (7) ➢ Good revenues/patient volume (13) ➢ Financial support (8) ➢ Increase in physicians’ income (25, 30) ➢ Guarantee of a position (12) ➢ Opportunities to perform research (12) ➢ Financial compensations (26) ➢ Loan repayment (29) ➢ Scholarship throughout their medical education (29) ➢ Wages (36) ➢ Future tuition (36) ➢ An alternate payment plan (39) ➢ Comprehensive payment plans (39) ➢ Increased funding (39) ➢ Direct financial support for overhead expenses (39) ➢ Special funding for rural clinics, including facilities, support staff, and administration, regardless of compensation model (39) ➢ Financial support for travel and accommodation (39) ➢ Financial motivations (40) ➢ Funding for continuing medical education (45)	➢ Having a partner who wants to live and work in a metropolitan environment (18) ➢ Desire for a metropolitan lifestyle (18) ➢ Unsuitable requirements of salary (16) ➢ Irregular payments (16) ➢ High deductibles (16) ➢ Lower financial bonus (7) ➢ Lower income (7) ➢ Willingness to pay for working in an urban area (7) ➢ Low remuneration (8) ➢ High tuition fees (8) ➢ Lack of economic incentives (26) ➢ Differences between private and public sector payments (26) ➢ Lack of rewards (27) ➢ supplementary income from locum (27) ➢ Economic problems (32) ➢ High fuel costs (45) ➢ Inappropriate payment (33) ➢ Inappropriate salary (33) ➢ Lower reimbursements (34) ➢ Influence of monitoring score on salary (35) ➢ Delay in payments (35) ➢ Low salary(37)	➢ Income sources (22) ➢ Income expectations (26) ➢ Financing Method (32) ➢ Monthly payment time (33) ➢ Rate of payment (37) ➢ Income (41)
**Career and professional factors**	➢ Group practice arrangement with other clinicians (18) ➢ Opportunities for leadership (18) ➢ Job growth opportunities (19) ➢ Working toward health equity (4) ➢ Promotion prospects status (20) ➢ Providing facilities for phone or email contact with seniors or specialist colleagues for advice (20) ➢ Career development (20) ➢ Professional development opportunity (20) ➢ Functional teams (20) ➢ Autonomy on clinical issues (14) ➢ Professional support (22) ➢ Career opportunities (22) ➢ High professional expectations (22) ➢ Training opportunities (7) ➢ Skills Development (7) ➢ Longer work experience (7) ➢ Scholarship obligation (13) ➢ Autonomy/freedom in the rural practice setting (13) ➢ Recruiting and developing students with an ‘affinity’ for rural communities and services (13) ➢ Educational strategies (8) ➢ Reservation of post-graduate seats for doctors who have served in rural areas (8) ➢ Involving rural doctors in teaching medical students (8) ➢ Arranging continuing education programs for rural doctors (8) ➢ Introduction of compulsory rural service after graduation (8) ➢ Educational investment (8) ➢ Offering professional development opportunities (8) ➢ Educational initiatives to create doctors for rural areas (8) ➢ Selecting medical students who have a rural background (8) ➢ Community-based medical Education (8) ➢ Providing a partial or complete tuition fee waiver in return for rural service (8) ➢ Career Development (27) ➢ Professional or career incentives (27) ➢ Chance to learn new procedures (43) ➢ Rural or underserved postgraduate training (29) ➢ Retainer schemes (29) ➢ Continuing medical education workshops (29) ➢ Initiative Zone Educational Incentive scheme (29) ➢ Financial investment (29) ➢ Graduate in family medicine (31) ➢ Early rural exposure for trainees (45) ➢ Training local students (45) ➢ Educational opportunities (45) ➢ Increasing the number of rural students in local schools (45) ➢ Increasing admissions for rural students (45) ➢ Training and education (36) ➢ Training and purchase of patient-record and information systems (39) ➢ Providing forms of licensure to provide services (39) ➢ Provide forms of licensure to permit rural physicians to cross provincial borders to provide locum services (39) ➢ Career Development Programs (40) ➢ Absence of proper system or policy (40) ➢ Expanding education services (40) ➢ Provision of post-graduation and in-service training (40) ➢ Establishment of medical colleges in rural areas (40) ➢ Continuing medical education (41) ➢ National medical graduate (42) ➢ Accessing higher education (42) ➢ Emphasize on the social aspect of recruitment (42)	➢ Lack of collegial support among rural general practices (18) ➢ Lack of opportunity to take vacations for personal reasons or continuing medical education (18) ➢ High Professional standards (9) ➢ Specialty and educational requirements and infrastructure (22) ➢ Procedural skills (23) ➢ Shorter job history (7) ➢ Barriers to career advancement (13) ➢ Increasing specialization (25) ➢ Professional isolation (27) ➢ Not having continuing education opportunities (27) ➢ Professional imprisonment (27) ➢ Study leave or international opportunities (27) ➢ Lack of career development guidelines (27) ➢ Educational problems (32) ➢ Poor transportation infrastructure (45) ➢ professional isolation (35) ➢ Lack of promotion opportunities (37) ➢ Feel negative to current job prospects (37) ➢ Seeking better career development (37) ➢ Lack of chance of promotion (37) ➢ Low education level (37) ➢ Limited professional ability (37) ➢ Limited or distant access to specialists and technological support (39) ➢ International medical graduate (42)	➢ Practice size and type (23, 41) ➢ Long term education (7) ➢ Lack of promotion opportunities (13) ➢ Having an established career path (25) ➢ improvement opportunities (28) ➢ Educational factors (28) ➢ Type of secondary education, and type of higher secondary education (28) ➢ working expertise (31) ➢ Enhanced skills programs (45) ➢ Longer postgraduate training (45) ➢ Midlevel practitioners (34) ➢ Age (37) ➢ Lack of opportunity to use the abilities (37) ➢ Medical training (42) ➢ Changing the medical education model (42)
**Working conditions factors**	➢ Low workload (18) ➢ Regular work hours (18) ➢ level of collegial support (18) ➢ Supervisor level of support (18) ➢ Improvements in information technology (18) ➢ Job flexibility (25, 34, 19) ➢ Supporting systems (19) ➢ Staff function as team (19) ➢ Supportive family and work/life balance (19) ➢ Supportive administration (19) ➢ Effective support staff (19) ➢ Social justice (19) ➢ Developing a locum service to allow rural doctors to take annual leave and sabbaticals (20) ➢ A reasonable level of infrastructure (20) ➢ Working security (16, 20) ➢ A secure working environment (20) ➢ Opportunities for continuing medical education (20) ➢ Compulsory services commitment (16) Educating native manpower (16) ➢ Workplace with a high level of progressive healthcare (9) ➢ Good working environment (9) ➢ Good infrastructure (9) ➢ Rich instrumental background (9) ➢ Reasonable workload (9) ➢ Incentive benefits (9) ➢ Good equipment (9) ➢ Access to medical information and consultations (9) ➢ Opportunities to achieve professional goals (9) ➢ Modifiable characteristics of work (21) ➢ Offering leadership opportunities (21) ➢ Promoting practice ownership (21) ➢ Coordinating cross-coverage arrangements (21) ➢ Professional work (21) ➢ Preference for a location (22) Lack of educational opportunities for children (22) ➢ Work commitments (22) ➢ High career pathways (34) Annual leave opportunities (23) ➢ Business structure (23) ➢ Paid locum relief (23) ➢ Good relations with the supervisor (7) ➢ Increasing level of the facility (7) ➢ Call coverage (13) ➢ Provision of improved working conditions (8) ➢ Improving living and working conditions and environment (8) ➢ Good service infrastructure (25) Educational incentives to promote working (12) ➢ Personal security at workplace (12) ➢ Access to online information technology for bibliographic search (12) ➢ Ability to use new cutting edge technologies (12) ➢ Access to specialist referral network via telephone and web (12) ➢ Adequate infrastructure at workplace (12) ➢ Access to high-complexity regional hospital to refer patients (12) ➢ Contact with medical technology (12) ➢ Rewards and promotion system (27) ➢ support or supervision (27) ➢ Access to the scholarships (27) ➢ ➢ No possibility for contacting a colleague for advice (27) ➢ Organizational incentives (27) ➢ Infrastructure development (27) ➢ Recruiting rural students (29) ➢ Support for professional development or research marketing (29) ➢ Wellbeing or peer support initiatives (29) ➢ Chances for advancement on this job (30) ➢ Appropriate work conditions (30) ➢ The chance to do something that makes use of their abilities (30) ➢ Competence of the manager in making decisions (30) ➢ Work stability (30) ➢ Employment relationship (31) ➢ Fixed office instructions (32) ➢ The specified working hours (32) ➢ Quantity and quality of feedback (32) ➢ Fewer working hours (45) ➢ Immigration policies (45) ➢ Broad scope of practice (45) ➢ Practice opportunities (30) ➢ Positive clerkship experiences (30) ➢ Specific practice opportunities such as teaching, hospital work (30) ➢ Lack of access to electronic medical records (30) ➢ Established relationships with specialists (30) ➢ Security at work (33) ➢ Change management (33) ➢ Political support of the government (33) ➢ Provide needed infrastructure (33) ➢ Organization of practice (35) ➢ Equipment (36) ➢ Social welfare benefits (37) ➢ Growth rate of investment in human resources (37) ➢ Devise retirement incentives specific to rural physicians (39) ➢ Clinical support program (39) ➢ Support staff (41) ➢ Ability to take time off (41) ➢ Group practice (41) ➢ No opportunity for working part-time	➢ Remaining located far from family and friends over an extended period (18) ➢ Less opportunity for private practice (19) ➢ Inadequate food service equipment (20) ➢ Minimal facilities (20) ➢ Lack of operating room facilities, equipment and blood transfusion services or even basic essential drugs (20) ➢ Mismatch between doctors’ skills and hospital facilities (20) ➢ Long working hours (16) ➢ Lack of job security (16) ➢ High working responsibilities (16) ➢ Local authorities’ bad behavior (16) ➢ Staff’s bad behavior (16) ➢ Improper facilities (16) ➢ Inefficient performance monitoring and evaluation (16) ➢ International medical school graduate (21) ➢ Restrictions on practice location (23) ➢ Lack of labor mobility among physicians (7) ➢ Distance from specialists and medical testing facilities (13) ➢ Adequate equipment (7) ➢ Hard work/long hours (13) ➢ Minimal laboratory and technical support (8) ➢ Lack of enough technological facilities (25) ➢ Socioeconomic characteristics of the designated areas (26) ➢ Income disparities between underserved areas and large cities (26) ➢ Inadequate accommodation facilities (26) ➢ Lack of recreational services (26) ➢ Higher workload (27) ➢ Lack of Mentoring (27) ➢ Absence of library facilities or technical resources (27) ➢ Lack of enough transportation facilities (27) ➢ Inability to use in-service training (27) ➢ Lack of clear incentives (27) ➢ Weak information systems (27) ➢ Systemic and organizational problems (32) ➢ Long working hours (33) ➢ Sharing on-call with only one other physician (34) ➢ Lack of job security (35) ➢ Low amenities (35) ➢ Long working hours (35) ➢ High work risk (37) ➢ Work pressure (37) ➢ Poor work condition (37) ➢ Lack of job safety (37) ➢ Lack the freedom to choose their own method of working (37) ➢ Amount of variety in the job (37) ➢ Industrial relationship between management and workers (37) ➢ Feeling of unsafe job (37) ➢ Continuing medical education (39) ➢ Limiting on-call duty (27) ➢ Compulsory services of doctors in rural health facilities (40) ➢ High ours worked and on-call frequency (40)	Work schedules (21) ➢ Workload (23) ➢ Work hours (23) ➢ Work culture (23) ➢ Hospital Infrastructure (27) ➢ Amount of work (30) ➢ Organization policies (30) ➢ Level of attention by leaders (30) ➢ Workload (45) ➢ Number of referrals for services (35) ➢ Poor work environment (37) ➢ Work hours (37)
**Personal factors**	➢ Supportive family (20) ➢ Self-care while working in high pressure settings (20) ➢ Political stability and security (20) ➢ Desirable family situations (21) ➢ having a personal and family rural background (18) ➢ Older age (22) ➢ Having a rural background (26, 22, 7, 31, 42, 45) ➢ Having a feel for the area (22) ➢ Perceptions of work life balance (23) ➢ Family ties to the community (13) ➢ Being able to help and feelings of patients’ appreciation of the physician (13) ➢ Good partners (13) ➢ Dependence on rural community (13) ➢ Place of residence (13) ➢ Exposure to rural practice before residency program (24) ➢ Marriage to a person from a rural area (24) ➢ Negative opinions about working in rural areas (25) ➢ Family physicians’ reluctance to change locations (25) ➢ Non-monetary incentive policies (25) ➢ Older age (12) ➢ Not having a university-trained professional parent (12) ➢ Previous exposure or service in a poor area (12) ➢ Willingness to practice medicine in rural or underprivileged areas (12) ➢ Interest in serving vulnerable people (12) ➢ Religious beliefs (26) ➢ Positive attitude towards working in remote areas (26) ➢ Lifestyle-related issues (26) ➢ Interest in serving rural setting (27) ➢ Less experience (27) ➢ Rural-origin graduates (28) ➢ Rural place of birth (28) ➢ Parent’s occupation (28) ➢ Occupation of father (28) ➢ Rural health related work experience (29) ➢ Family ties to the region (45) ➢ Residency experiences (30) ➢ knowledge gained during residency of the local community programmers (30) ➢ Being native (33) ➢ Job opportunities for spouses (39) ➢ Work-life balance (41) ➢ Professional interaction (41) ➢ Rural training (41) ➢ Spousal employment opportunities (41) ➢ Importance of finding spousal employment (42) ➢ Raising a family in a rural environment (42) ➢ Alignment of personal culture and skill set with that in the community or practice (42)	➢ Unsatisfactory rural elective experiences (18) ➢ Family pressure (16) ➢ Having high professional expectations (22) ➢ Placing a high value on prestige (22) ➢ Being bonded and clinical experience (22) ➢ Family and friend closeness (22) ➢ Seeking new employment (7) ➢ Wish to change employment (7) ➢ Being female (12) ➢ Anxiety about the unfamiliarity of rural life (27) ➢ The turnover intention (30) ➢ Being away from family (35) ➢ Willingness to continue education (35) ➢ Spousal and family concerns (39) ➢ Considerations of children (39)	➢ Personal and professional characteristics (21) ➢ Gender (22) ➢ Specific demographic (23) ➢ Gender (23) ➢ Age (23) ➢ Parental education levels (7) ➢ Birthplace (24) ➢ Year of graduation (24) ➢ The rank of the university attended (24) ➢ Place of residence (24) ➢ Marital status (26, 31) ➢ Individual preferences (26) ➢ Number of children (31) ➢ Year of University entrance (31) ➢ Age (31, 39, 42, 45) ➢ Gender (26, 24, 31, 42, 32, 39, 42, 45) ➢ Family member (37) ➢ Spouse’s career (42)
**Cultural factors**	➢ Gaining an understanding of the needs of people living in rural areas (18) ➢ Patient communication (19) ➢ Patient trust (19) ➢ Quality of doctor patient relationships (14) ➢ Nice people or community (13) ➢ Values (26) ➢ Personal incentives (27) ➢ Target population culture (32) ➢ Deep mutual trust and communication (37) ➢ Lack of social networks and investment in the community (38)	➢ The misconception that rural practitioners are less qualified than urban specialists (18) ➢ Poor culture of people (16) ➢ Inappropriate treatment of people (33) ➢ Social problems (37) ➢ Cultural differences (38) ➢ Lack of cultural opportunities (42) ➢ High cultural expectations (42)	➢ Degree of respect and recognition (27) ➢ Relationship with patients (37)
**Living conditions factors**	➢ Suitable housing and access to facilities (18, 23) ➢ Completion of satisfactory electives in rural areas (18) ➢ Provision of adequate schooling for their children or scholarships for boarding school (20) ➢ Jobs for spouses (9) ➢ Suitable places for their children in schools (9) ➢ free housing or subsidized house purchases (9) ➢ Opportunities to enjoy outdoor sports (14) ➢ Access to cultural activities (14) ➢ Lifestyle goals (22) ➢ Having a placement in a rural area (22) ➢ Free housing (25, 7) ➢ Lack of private paying patients (13) ➢ Lack of shopping or restaurants (13) ➢ Access to good schools for children (24) ➢ Social and recreation opportunities (30) ➢ Services in the area and lifestyle opportunities afforded by the location (30) ➢ Education facilities for children (36) ➢ Fit with the community; spouse/partner fit with the community (38) ➢ Appropriate relationships with individuals/community (38) ➢ Attractiveness of a rural location to the spouse (39) ➢ Recreational opportunities (39) ➢ Housing availability (41) ➢ Community incentives (41) ➢ Adequate housing availability (41) ➢ Strong education system for children (42) ➢ Safety of the community (42) ➢ Access to recreational facilities (42) ➢ Activities and proximity to an urban center (42) ➢ Proximity of the location for work and personal lives in a rural environment (42)	➢ Few social and recreational activities to enjoy (18) ➢ Inadequate accommodation facilities (18) ➢ Separation from partner and family (18) ➢ The absence of phone networks and other forms of communication even post (20) ➢ Lack of opportunities for a social life (20) ➢ Lack of vacation requirements or vacation leave (16) ➢ Region insecurity (16) ➢ Distrust to local authorities (16) ➢ Region depravity (16) ➢ Partner and family considerations (22) ➢ Geographic location (23) ➢ Limiting food choices (8) ➢ Unavailable Telephone services (8) ➢ Social and cultural problems (32) ➢ Environmental problems (32) ➢ Welfare problems (32) ➢ Inappropriate behavior of the people of the area (35) ➢ Political differences (38)	➢ Size of town ➢ Higher socioeconomic background (7) ➢ Wealth ranking (28) ➢ Population covered area (33) ➢ population growth and urbanization progress (37) ➢ Community size (39) ➢ Current community of residence (42) ➢ Able to grow in [a supportive] environment (42)

## Discussion

In this study, factors governing the retention of physicians in rural and underdeveloped areas were identified through a systematic literature review. According to our findings, the following six major categories govern the retention of physicians in the mentioned areas: financial, professional, working condition, living condition, cultural, and personal factors.

### Financial factors

Most of the studies had examined the desertion and retention of physicians in rural and underdeveloped areas mainly based on financial factors, such as income, salary, loans and appropriate reimbursement. One of the major incentives for most of the physicians is their revenue. Many of them neglect the well-being of cities in order to have more income, although this is not the only motivational factor. Physicians who were employed in underdeveloped and remote areas had numerous complaints regarding their low income, reimbursements and compared them with their counterparts in the urban areas.

Financial rewards and incentives have a significant effect on the desertion and retention of physicians in the aforementioned areas. It makes sense for people to prefer urban and well-developed areas to rural in terms of equal income.

Two studies reported that the inappropriateness of income rates and interrupted payments cause the physicians’ reluctance to serve in rural areas [16, 20]. In most of the studies, salary was a factor significantly affecting the retention rate in rural and underserved areas [46, 47]. Furthermore, the physicians in rural and deprived areas had fewer opportunities for working in private sector and seemed to be less satisfied with their incomes [23]. A study by Colleen Morken et al. (2018) showed that loan repayment was the least important factor in physicians’ retention in rural areas [48]. Pathman and Konrad (2004) indicated that economic vitality of communities attracted physicians. However, it did not influence physicians’ subsequent retention [21]. The result of another in Turkey demonstrated that an increase in physicians’ income reduced their willingness to work in rural areas [25]. Thus, the solution to this challenge is to increase the physicians’ salaries or to change reimbursement methods [26].

### Career and professional factors

Providing educational and skill improvement opportunities was another factor in the satisfaction of physicians who served in deprived areas [5]. Nonetheless, there was no public agreement on it [26]. Medicine is a science that needs to be updated based on the newest validated information and knowledge. Although there are many possibilities for being updated in today’s world, these facilities are less accessible in rural areas. Some physicians, who worked in rural and deprived areas, argued that activities in rural areas prevented them from developing professional skills [25]. According to the evidence, educational factors were the most effective factors determining the physicians’ choices of workplace [34]. Therefore, the relevant educational and professional policies should be considered. These policies include reserving the post-graduate seats for physicians who have worked in remote areas, providing the physicians in rural areas with the opportunity to teach certain courses at universities of medical sciences, and arranging continuing educational programs for rural physicians [8]. On the other hand, the results of Colleen Morken’s study (2018) showed that professional development opportunities and opportunities to teach medical students or residents’ courses did not have a great effect on the physicians’ retention in rural areas [48].

### Working conditions factors

Our findings showed that working conditions factors are the factors that have significant influence on the retention of physicians in rural or under development areas. The working conditions factors have been mentioned in most of the studies.

In some studies, working conditions, environment, and opportunities for professional improvement were mentioned as affecting factors on physicians’ recruitment and retention in rural areas [44, 49]. Our study showed that unavailability of surgical equipment and the lack of essential drugs are discouraging factors in the physicians’ work in the rural and underdeveloped areas [50]. In other words, the developmental condition of the region, where the physicians worked, was a very important variable adversely shaping their preferences [25]. The lack of job security, heavy workload, length of working hours, and the multiplicity of the physicians’ responsibilities in rural health centers were among the other important factors in physicians’ unwillingness to stay in the deprived areas.

In Vietnam, there was a distinct lack of job replacement among physicians. The physicians who worked at a certain level of an organization could only be transferred within the same region and geographical area from one level to another level in other organizations [5].

In rural areas, usually, there is not any medical specialist that could help or guide other physicians specially GPs. Thus, they should visit patients and make decisions by themselves at times even without any preclinical exams or have to work seven days of the week.

Job security and the possibility of relocation were introduced in the Shankar’s (2010) review as factors in the retention of physicians [8].

Evidence showed that there was a strong relationship between the physicians’ occupational group and choice of workplace. For example, specialists who worked in hospitals with access to advanced technologies were reluctant to work in rural or underdeveloped areas where technological facilities were meager [25]. Therefore, it was stated that an increase in specialization contributed significantly to the imbalance in the geographical distribution of physicians [51].

### Personal factors

Personal factors were introduced in a limited number of studies [9] as influencing factors in physicians’ retention in deprived areas. Prasad and Amatya (2015) argued that the personal factors including demographic factors are more significant than the financial factors [28]. Among the demographic factors, gender and age had the greatest impact In general; male physicians’ tendency to serve in rural and underdeveloped areas was greater than the female physicians [12, 26]. This issue may stem from the fact that female physicians harbor more concerns on workplace conditions, and their choices are often more affected by factors such as security issues, responsibilities, family commitments, living conditions and life considerations in comparison to their male colleagues [25, 52].

In the literature, the findings concerning the effect of age on working in rural and underdeveloped areas are contradictory. According to Mollahaliloglu (2015), young physicians were the groups that could be easily motivated to work in rural areas [25]. However, most of the studies showed that older physicians had a greater tendency to serve in deprived areas in comparison with the younger ones [12, 22]. It can be argued that younger physicians often place a higher value on education, career progression, and leisure activities compared to older physicians.

According to Joseph Lee)2016(, family ties were one of the factors which influenced the physicians’ decision on retention in rural areas [44]. Therefore, the selection of physicians based on their readiness and desire to work in underdeveloped areas as a result of either interest or family ties in these areas can enhance their recruitment and retention in underdeveloped areas.

The physicians, who were from the ethnic communities in rural areas along with the physicians who were born and raised in rural places (i.e., had a rural background), were more inclined to serve in rural and underdeveloped areas [24]. One possible explanation is that these physicians were more familiar with the deprived environments and were able to easily adapt themselves to the conditions and problems of the deprived environments. Furthermore, the physicians, who passed their courses in the deprived areas or had a record of serving in deprived and rural areas, tend to serve in these areas more readily in comparison with other physicians without such backgrounds. This issue stems from the fact that during their studies, these physicians had seen and experienced the living conditions in the rural environments and have kept preparing themselves to deal with such problems [2, 34]. Similarly, the findings of a number of other studies confirmed that rural background, rural origins, and rural lifestyle were the factors highly associated with recruitment and retention of health professionals in rural areas [12].

Some physicians enjoy serving in deprived areas because they had a high motivation to treat the patients who were in need of help [13]. In other words, these physicians were happy to serve in deprived areas because they believed that their efforts helped to establish a mutual understanding among community and society members.

### Living conditions factors

Deprivation rates of an area had a direct impact on physicians’ tendency to stay in their workplaces or to leave them. The deprivation severity of the region was a major factor in the physicians’ desertion or decisions on the desertion of their workplaces in the following years [53].

Lack of amenities, accommodations, and some deficiencies such as lack of communication and internet systems, recreational facilities and interesting places prompted the physicians to leave their workplaces [14, 54].

In some cases, long distances between the rural areas and the urban centers restricted the physicians’ access to shopping centers and stores which were necessary to buy their required food and clothing items. These factors made the physicians reluctant to continue to serve in these areas [13]. Furthermore, the physicians, who worked in the rural areas, faced a higher risk in terms of health and safety in comparison with the physicians who worked in the urban areas. More specifically, the risk of certain diseases was high in rural and remote areas due to the fact that people disregarded some health issues [5]. Evidence showed that lifestyle opportunities, including the availability of cycling opportunities, parks and restaurants together with adequate recreation affected the retention of physicians in rural areas. Therefore, providing the physicians with recreational opportunities in underdeveloped and rural areas should be taken into consideration [18, 44].

### Cultural factors

A limited number of studies have addressed cultural factors in the retention of physicians. The customs, traditions, beliefs, moral values, language, and laws of the rural community can help GPs to work in rural areas for a long period of time, but in some cases, they could be counterproductive.

The existence of appropriate bilateral relationships between physicians and patients was one of the important issues in these areas and could lead to these physicians’ satisfaction and retention in deprived areas [33]. A number of physicians reported the people’s breach of etiquette in deprived and rural areas [22]. This issue could stem from the people’s poor living conditions and their lack of awareness of the rules and conventions of the etiquette in health centers. Therefore, it is necessary to develop a framework and code for communication between the physicians and their patients in order to increase the physicians’ motivation to serve in disadvantaged areas [28]. The government policy makers and universities of medical sciences have a major effect on the culture of working in limited-resource and underprivileged areas of the countries [12].

### Strengths and limitations

To the best of our knowledge, this is the first study of its kind to systematically explore all the factors governing the retention of physicians in underdeveloped or deprived areas in both developed and developing countries published papers, although we only included papers that were written in English. Strength of this review is conducting a systematic search in five databases, supplemented by a hand search of the bibliography in the articles identified. This review is limited by the difficulty of analyzing, identifying the category of some extracted factors and comparing findings of studies conducted in a wide diversity of contexts, with a variety of using different study designs.

## Conclusion

This study demonstrates that there are different factors influences the physicians’ interest in rural and remote areas and their retention in these areas. A good number of studies over this issue have been conducted in developing and developed countries. Working conditions factors and financial factors deserve healthcare policy makers’ particular attention among the factors which are associated with the retention and willingness of physicians to serve in deprived areas. Recruiting physicians, who are from rural backgrounds and rural origins, is another determining factor in physicians’ retention which has to be considered by the policy makers who aim to promote the physicians’ retention in rural areas. There is no enough evidence regarding the cultural factors and their effect on the physicians’ retention in the mentioned areas. Further studies are warranted to determine the quantitative effects of the identified determining factors on the retention of physicians in rural and underdeveloped areas.

Identifying the influential factors can be useful to policy makers and health planners who intend to develop evidence-informed policy interventions and executive programs in order to guarantee the retention of the healthcare workers in these areas which is a crucial factor in the improvement of population health.

## Supplementary information

**Additional file 1: Table 1.** PubMed search strategy.

## Data Availability

The datasets used and/or analysed during the current study are available from the corresponding author on reasonable request.

## Abbreviations

HCW: Health Care Workers
GP: General Practitioner
CASP: The Critical Appraisal Skills Program
