# Supplementary material for: Determining factors in the retention of physicians in rural and underdeveloped areas: a systematic review
Source: BMC Fam Pract. 2020 Oct 23;21:216. doi: 10.1186/s12875-020-01279-7 (PMC7585284; doi:10.1186/s12875-020-01279-7)
Supplement: Supplementary file 1 — Additional file 1: Table 1. PubMed search strategy. [file 12875_2020_1279_MOESM1_ESM.docx]

| Appendix table 1: PubMed search strategy |
| --- |
| ((((((“poor area”[Title/Abstract] OR “marginal area”[Title/Abstract] OR “underserved area”[Title/Abstract] OR “slum area”[Title/Abstract] OR “rural area”[Title/Abstract] OR “suburban area”[Title/Abstract] OR “remote area”[Title/Abstract])) OR (“poor region”[Title/Abstract] OR “marginal region”[Title/Abstract] OR “underserved region”[Title/Abstract] OR “slum region”[Title/Abstract] OR “rural region”[Title/Abstract] OR “suburban region”[Title/Abstract] OR “remote region”[Title/Abstract])) OR (“poor setting”[Title/Abstract] OR “marginal setting”[Title/Abstract] OR “underserved setting”[Title/Abstract] OR “slum setting”[Title/Abstract] OR “rural setting”[Title/Abstract] OR “suburban setting”[Title/Abstract] OR " remote setting”[Title/Abstract]))) AND (“willingness to work”[Title/Abstract] OR “intention to stay”[Title/Abstract] OR “intention to leave”[Title/Abstract] OR desertion[Title/Abstract] OR retention[Title/Abstract] OR motivation[Title/Abstract] OR sustain*[Title/Abstract] OR recruitment [Title/Abstract] OR “service delivery”[Title/Abstract] OR “service provision”[Title/Abstract] OR stay[Title/Abstract] OR work[Title/Abstract])) AND (doctor*[Title/Abstract] OR physician*[Title/Abstract] OR specialist*[Title/Abstract] OR “general practitioner”[Title/Abstract] OR general physician*[Title/Abstract]) |
